# The Use of MRI With Double-Dose Eovist for the Detection of Bile Leaks

**DOI:** 10.7759/cureus.102780

**Published:** 2026-02-01

**Authors:** Megan L Halloran, Olivia Spaedy, Raymond I Okeke, Matthew Toedt, Justin Rehder, Ramy Shoela, Amirhossein Mohammadian Bajgiran, Hany Elbeshbeshy, Jeffrey Brown, Mustafa Nazzal

**Affiliations:** 1 Department of Obstetrics and Gynecology, Tufts Medical Center, Boston, USA; 2 Department of Obstetrics and Gynecology, Penn State Health Milton S. Hershey Medical Center, Hershey, USA; 3 Department of Surgery, Saint Louis University Hospital, St. Louis, USA; 4 Department of Internal Medicine, Saint Louis University Hospital, St. Louis, USA; 5 Department of Neurology, Saint Louis University Hospital, St. Louis, USA; 6 Department of Radiology, Saint Louis University Hospital, St. Louis, USA; 7 Department of Internal Medicine, Division of Gastroenterology and Hepatology, Saint Louis University Hospital, St. Louis, USA; 8 Department of Abdominal Imaging, Division of Diagnostic Imaging, University of Texas MD Anderson Cancer Center, Houston, USA

**Keywords:** bile leaks, endoscopic retrograde pancreatico-cholangiography, eovist, liver transplant, magnetic resonance imaging

## Abstract

Background and objectives

Bile leaks are serious complications that can occur after liver transplant, hepatobiliary surgery, and abdominal trauma and can be challenging to diagnose. Noninvasive diagnostic studies for bile leaks are limited. In this study, we aimed to evaluate the efficacy of MRI with a double-dose of Eovist (gadoxetate disodium) in comparison with other imaging modalities for detecting biliary leaks.

Methods

This study involved a retrospective review of patients who underwent MRI with double-dose Eovist (dEovist) to evaluate suspected bile leakage from 2016 to 2022. Indications for evaluation included suspected bile leak after recent hepatobiliary surgery or penetrating right upper quadrant trauma. Once the dEovist study was performed and a bile leak was suspected, confirmation with endoscopic retrograde cholangiopancreatography (ERCP) was obtained. These results were statistically analyzed to determine the sensitivity and specificity of MRI with dEovist in detecting biliary leakage, and ERCP was used as the confirmatory test.

Results

A total of 19 patients were included. dEovist detected biliary leakage in five of 19 patients. All patients with concern for bile leak after MRI underwent ERCP for confirmation of the findings; four of these patients were confirmed as true positives, and one was determined to be a false positive. The remaining 14 patients with negative MRI findings with dEovist were followed clinically, and all had clinical courses that were not suggestive of bile leak. Six of these patients underwent ERCP, which also confirmed the absence of biliary leakage. The sensitivity of MRI with dEovist for detecting bile leaks in this study was 100% (95% confidence interval (CI): 39%, 100%), and the specificity was 93% (95% CI: 66%, 100%).

Conclusions

Prompt identification and early intervention are crucial for preventing the complications associated with bile leaks. The use of MRI with dEovist has shown promising results as a noninvasive imaging modality with high sensitivity and specificity in the detection of bile leaks.

## Introduction

Bile leaks, although rare, pose serious complications after liver transplant, hepatobiliary surgery, and abdominal trauma and require urgent intervention to prevent further morbidity and mortality [1,2]. Bile leaks have an incidence rate of 0.8-1.1% in laparoscopic cholecystectomies, 2 to 25% in liver transplants, and 5% in liver resection [3]. In addition to impairing quality of life, extending the length of hospital stay, and increasing medical costs, complications of bile leak include fever, abdominal pain, sepsis, strictures, transplant failure, and death [3,4]. Research into sensitive and specific noninvasive methods to detect bile leaks is necessary to prevent complications associated with both bile leakage and invasive imaging methods.

Prompt identification and early intervention are crucial for preventing these complications; however, there is no established standard protocol for detecting bile leaks. The current imaging methods used to detect bile leaks are CT, Doppler ultrasound (US), cholangiography, cholescintigraphy, hepatobiliary iminodiacetic acid (HIDA) scan, MRI, magnetic resonance cholangiopancreatography (MRCP), and endoscopic retrograde cholangiopancreatography (ERCP) [3]. Although these modalities can provide evidence of a bile leak, the findings have been shown to be nonspecific. Because of this limitation, Eovist, a gadolinium-based MRI contrast agent, represents a promising imaging method for more sensitive and specific detection [5].

Gadoxetic acid (Gd-EOB-DTPA), known by the trade names Eovist in the US and Primovist in Europe and Australia, is a hepatobiliary-specific contrast agent. Eovist is particularly efficacious in liver imaging due to the presence of ethoxybenzyl groups, which are selectively taken up by hepatocytes and enhance normal liver parenchyma on T1-weighted images [5]. The pharmacokinetics of Eovist are similar to those of other liver-specific contrast agents, such that in the early phase, information is provided about liver lesion vascularity. In the delayed phase, information is provided about hepatocyte presence and function [6]. This dual capability gives Eovist the advantage of having both dynamic imaging capability and delayed hepatobiliary phase imaging.

In addition to being taken up by hepatocytes, Eovist is also excreted in the bile, thus expanding its imaging applications. In humans, approximately 50% of an administered Eovist dose is transported through hepatocytes and excreted into the bile, compared to approximately 5% biliary excretion of the next strongest hepatobiliary contrast agent, gadobenate dimeglumine [6]. Preoperatively, Eovist has the ability to detect intrahepatic ducts and anatomical variants that conventional T2-weighted cholangiopancreatography might not. Confirming these variations can be key to reducing the risk of biliary leak via inadvertent transection, a major cause of morbidity in liver transplantation [3]. Postoperative contrast-enhanced imaging is used to detect any ligation or stricture of the biliary system that might have occurred as a result of the procedure, as well as to detect any biliary leaks [7].

Although this is a promising technique for diagnosing bile leaks quickly and effectively, there has been limited research on the use of MRI with double-dose Eovist (dEovist) in this setting. Double dosing of Eovist has been shown to improve image quality compared with single doses in cardiac MRI [8]. Additionally, double-dose Eovist was found to provide better arterial enhancement of hepatocellular carcinomas and to improve lesion-liver contrast in the hepatocyte phase [9]. However, there is currently no research specifically evaluating the use of MRI with dEovist for the detection of bile leaks, and this study aims to address this gap by exploring this modality.

The abstract of this article was previously presented as a poster at the American Transplant Congress 2024 on June 2, 2024.

## Materials and methods

Patient demographics

Approval was obtained from the institutional review board to conduct this retrospective review. A total of 21 patients who underwent MRI with dEovist between January 2013 and January 2022 were identified. Among these patients, two were excluded due to inaccessible medical records. Patient history, hospital presentation, initial modality suggesting bile leak, and MRI with dEovist interpretation were recorded for each patient. Patient charts were analyzed to identify the use of different imaging modalities for detecting biliary leakage, and those performed before MRI with dEovist were recorded.

Imaging technique

The sequence protocol for patients undergoing MRI for the detection of biliary leaks includes both T2-weighted magnetic resonance cholangiography (T2w-MRC) and contrast-enhanced MRC (CE-MRC). Studies are performed using a 16-channel phase array body surface coil and a 3T MRI (MAGNETOM Skyra; Siemens Healthcare, Berlin, Germany) or a 1.5T MRI (MAGNETOM Aera; Siemens Healthcare).

T2w-MRC is acquired using half-Fourier acquisition single-shot turbo spin echo (HASTE) sequences in transverse and coronal planes. Twelve 50-mm thick sections were then acquired in oblique coronal planes in the course of the bile duct (as seen in the axial images) using a field of view of 300 mm. 3D-SPACE (sampling perfection with application optimized contrasts using different flip angle evolutions) were also obtained in 1-mm thickness using a field of view of 380 mm. For optimal visualization of ducts, acquired images are reformatted in different planes using multiplanar reconstruction (MPR) and maximum intensity projection (MIP).

CE-MRC is acquired using breath-hold three-dimensional (3D) fat-suppressed gradient echo (GRE) T1w VIBE (volumetric interpolated breath-hold examination) sequence during the arterial, portal venous, equilibrium, and hepatocyte phases after intravenous administration of 0.2 ml/kg (0.50 mmol/kg) Gd-EOB-DTPA (Eovist, Bayer HealthCare, Tarrytown, NY) at a rate of 2 ml/s, followed by a bolus of 20 ml saline. Hepatocyte phase images are routinely acquired approximately 5, 10, 20, and 60 minutes after contrast administration for the detection of the biliary extravasations. The flip angle of a 3D gradient-echo T1- weighted sequence is 9°. Additional T1w VIBE images are obtained with a flip angle of 30° to improve contrast between the bile duct and liver in 20 and 60 minutes hepatocyte phase because of the increased T1- weighting of the pulse sequence.

The standardized protocol used to perform is presented in the following table (Table 1).

**Table 1:** Standardized MRI protocol for detecting bile leaks MRI: magnetic resonance imaging; T2w-MRC: T2-weighted magnetic resonance cholangiography; CE-MRC: contrast-enhanced magnetic resonance cholangiography

Sequence/parameters	Orientation	Field strength (T)	TR (ms)	TE (ms)	Flip angle (°)	Field of view (mm)	Matrix	Slice thickness (mm
T2w-MRC	Transversal	3	1400	95	160	328 x 420	200 x 320	6
T2w-MRC	Coronal	3	1610	91	160	400 x 400	288 x 320	6
3D-SPACE	Transversal	3	2400	698	125	1380 x 380	353 x 384	1
CE-MRC	Transversal	3	3.37	1.3	9	328 x 420	188 x 320	2.5

Statistical analysis

Sensitivity and specificity were calculated with the following formulas, respectively: (True Positive) / (True Positive + False Negative); (True Negative) / (True Negative + False Positive). A 95% confidence interval (CI) was calculated for both sensitivity and specificity by using a Z score of 1.96.

## Results

Patient demographics

A total of 19 patients were evaluated using MRI with dEovist for bile leak (Table 2). Patient age ranged from 38 to 75 years, with 13 male patients and six female patients. Patient BMI ranged from 22.0 to 55.7 kg/m^2^. Common presentations prompting concern for biliary leakage included elevated liver enzymes in 21% of patients and post-liver transplant follow-up in 21% of patients. MRI with dEovist was used to evaluate biliary leak detection in all patients, and all positive MRI results were confirmed for bile leak with ERCP. All patients with negative MRI results were followed to assess for clinical courses concerning a bile leak.

**Table 2:** Summary of patient characteristics Y: yes; N: no; M: male; F: female; GSW: gunshot wound; RUQ: right upper quadrant; LFT: liver function test; ALP: alkaline phosphatase; CBD: common bile duct; CT A/P: CT abdomen/pelvis; US: ultrasound; HIDA: hepatobiliary iminodiacetic acid; TACE: transarterial chemoembolization

Patient	Age (years)	Sex	BMI (kg/m^2^)	Presentation to the hospital	Prior cholecystectomy (Y/N)	Prior liver Tx (Y/N)	Recent ERCP (Y/N - value)	Initial imaging after presentation (before MRI w/ Eovist)	Eovist dose (mL)
1	61	M	28.2	Post-liver transplant hyperkalemia, elevated LFTs, and hyperbilirubinemia	N	Y	N	CXR, liver biopsy	20
2	41	F	26.7	Liver transplant	N	Y	Y	US, CT A/P	15
3	38	M	N/A	Abdominal pain s/p GSW	N	N	N	US, CT A/P, MRI w/o Eovist	10
4	47	F	27.0	Melena and abdominal pain	Y	N	N	CT	15
5	75	M	24.6	Liver transplant	N	Y	Y	HIDA	20
6	45	F	55.7	Concern for CBD injury	Y	N	Y - 2	None	20
7	56	M	31.8	Sharp pain around the 12th rib	N	Y	N	CT A/P	17
8	47	M	30.1	Chills and fever	N	Y	Y - 3	CT A/P	10
9	60	F	28.4	Abdominal pain	Y	N	N	Renal transplant sonogram, CT w/o contrast	15
10	68	M	N/A	Converted lap: cholecystectomy to open	Y	N	Y - 2	None	20
11	64	M	29.0	Elevated ALP post-liver transplant	Y	Y	Y	CT A/P w/ contrast	20
12	56	M	28.4	Bile tinging of JP drain, abdominal pain, and distension s/p pancreaticoduodenectomy	Y	N	Y - 1	CT A/P w/ contrast	17
13	46	F	22.0	Elevated liver enzymes	Y	Y	Y - 4	None	12
14	57	F	23.5	Elevated LFTs post-liver transplant, TACE, and terminal ablation	N	Y	Y	US	12
15	61	M	41.5	Chills, fever, RUQ pain	Y	N	N	CT	20
16	70	M	N/A	Hepatic abscess	N	N	N	CT w/ contrast	15
17	57	M	34.8	RUQ pain	Y	N	N	None	20
18	53	M	31.4	Liver transplant follow-up	N	Y	N	None	20
19	63	M	22.5	Chronic cholecystitis w/ abdominal pain	Y	N	N	CT	20

Imaging results

**Figure 1:**
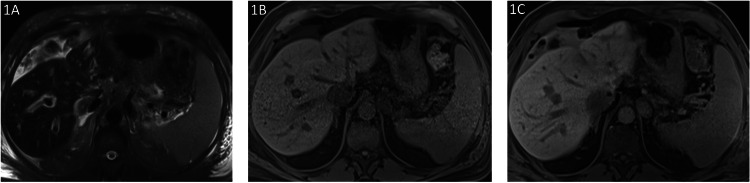
Imaging findings of a 75-year-old male: post-op day 7 after orthotopic liver transplant Axial T2 FS HASTE showed moderate volume T2 hyperintense perihepatic fluid (1A). Pre-contrast and 1 hour post-contrast axial vibe FS showed delayed enhancement of the perihepatic fluid consistent with biliary leak (1B and 1C). The source of the bile leak could not be definitively determined on imaging. ERCP performed the next day demonstrated no bile leak HASTE: half-Fourier acquisition single-shot turbo spin echo; ERCP: endoscopic retrograde cholangiopancreatography

**Figure 2:**
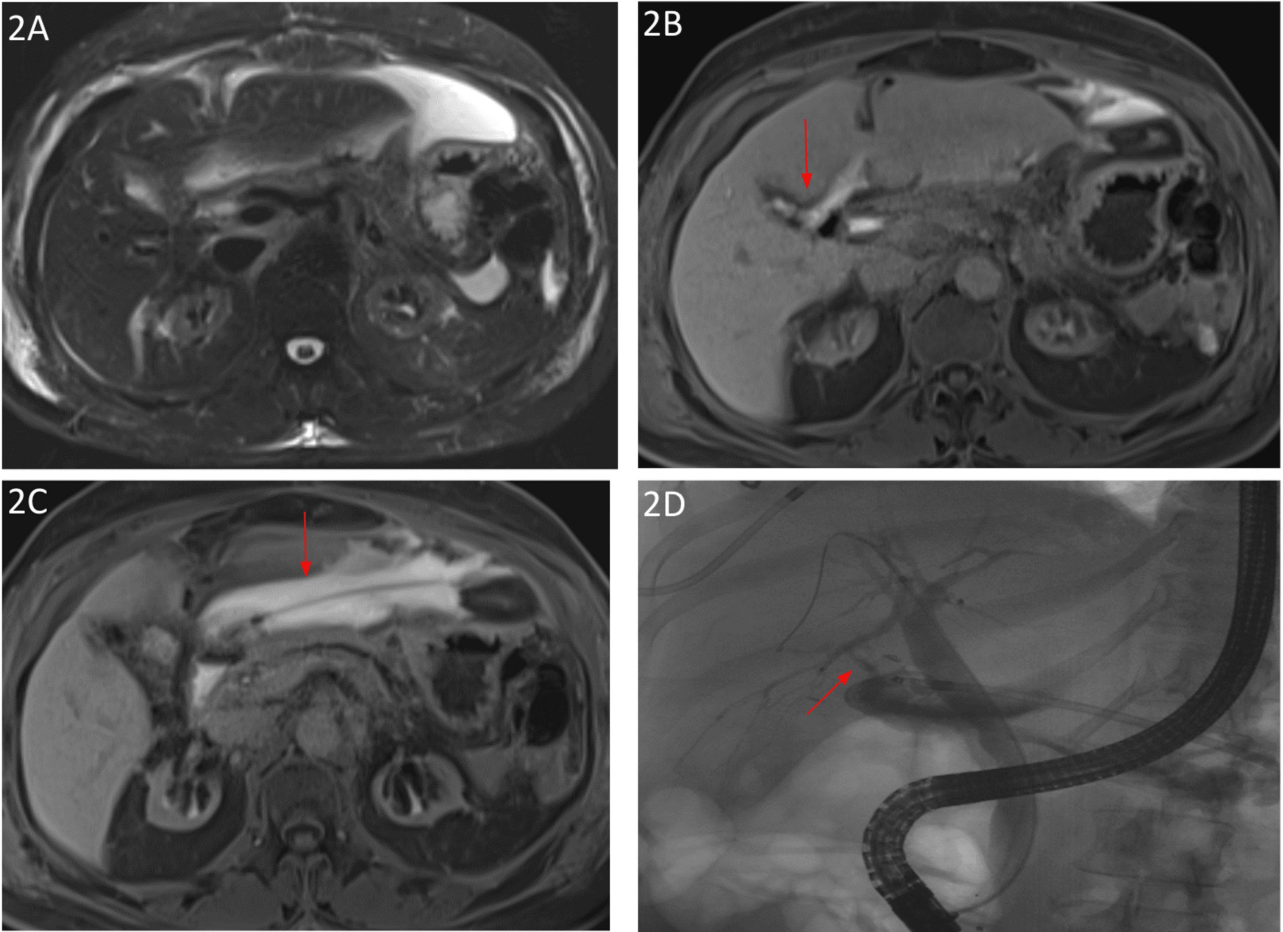
Imaging findings of a 60-year-old woman: postoperative day 14 status post laparoscopic cholecystectomy Complicated by a perihepatic fluid collection (2A). One-hour delayed axial vibe images demonstrate a bile leak from the right posterior biliary tree (2B arrowhead) with enhancing perihepatic biliary fluid (2B, 2C). Biliary ascites was confirmed on aspiration, and an active leak was confirmed on ERCP (2D arrowhead) ERCP: endoscopic retrograde cholangiopancreatography

**Figure 3:**
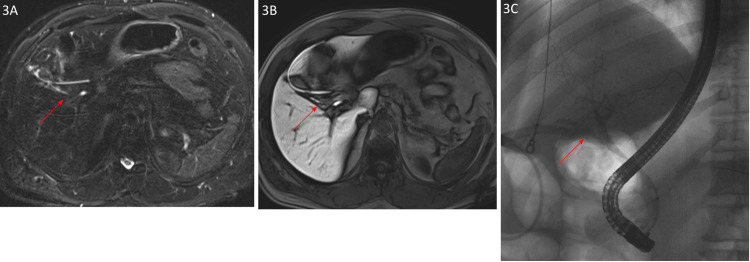
Imaging findings of a 57-year-old male: postoperative day 2 status post laparoscopic cholecystectomy (presenting with abdominal pain) MRCP demonstrates linear fluid collection in the gall bladder fossa extending from the cystic duct stump. Note the surgical drain in place (3A). One-hour delay axial vibe MRI image demonstrates linear enhancing fluid extending from the cystic duct stent, consistent with active biliary leak. (3B). ERCP image confirming active leak from the cystic duct stent (3C) MRCP: magnetic resonance cholangiopancreatography; MRI: magnetic resonance imaging; ERCP: endoscopic retrograde cholangiopancreatography

Double-dose Eovist detected biliary leakage in five of the 19 patients (Table 3). Fourteen of these patients had undergone various imaging before MRI with dEovist. All patients with concern for bile leak after MRI underwent ERCP for confirmation of results - four of these patients were confirmed as true positives (Figures 2, 3) and one was determined to be a false positive (Figure 1). The remaining 14 patients with negative MRI with dEovist were followed up clinically, and all had clinical courses that were not suggestive of bile leak. Six of these patients underwent ERCP, which also confirmed the absence of biliary leakage. The sensitivity of MRI with dEovist to detect bile leaks in this study was 100% (95% CI: 39%, 100%), and the specificity was 93% (95% CI: 66%, 100%).

**Table 3:** Summary of imaging results MRI: magnetic resonance imaging; Y: yes; N: no; ERCP: endoscopic retrograde cholangiopancreatography; HCC: hepatocellular carcinoma; CBD: common bile duct; S/p: status post

Patient	Initial imaging performed (Y/N)	Bile leak detected on initial imaging (Y/N/inconclusive/not performed)	Bile leak detected on MRI w/ Eovist (Y/N/inconclusive)	Final diagnosis
1	Y	N	N	Large right pleural effusion, a small left pleural effusion, and stenosis of the proximal hepatic artery
2	Y	N	N	Mild periportal edema, delayed biliary excretion, small-to-moderate right pleural effusion with associated right lower lobe atelectasis
3	Y	N	N	Possible noncommunicating biloma or evolving seroma/hematoma
4	Y	Inconclusive	N	Abdominal ascites due to presumed cirrhosis
5	Y	N	Y	MRI with dEovist showed a bile leak at the biliary anastomosis; ERCP the next day showed no bile leak
6	Y	N	Y	Common bile duct injury w/ bile leakage and mild diffuse hepatic steatosis
7	Y	Inconclusive	N	Abscess, biloma, or atypical HCC
8	Y	Inconclusive	N	Fluid collection in the left lobe of the liver and fatty infiltration around hepatic v. and branches
9	Y	N	Y	Evidence of bile leak with fluid collection from the perihepatic space to the gallbladder fossa. Possibly arising from the anterior branch of the R hepatic duct
10	N	Not performed	Y	Bile leak with mild diffuse hepatic steatosis
11	N	Not performed	N	Mild intrahepatic bile duct dilation with possible seroma
12	Y	N	N	Pancreatic leak
13	N	Not performed	N	CBD stricture and biliary/jejunal fistula
14	Y	N	N	Biliary anastomotic stricture
15	Y	Inconclusive	N	Gastritis
16	Y	N	N	Hepatic abscess leading to sepsis
17	N	Not performed	Y	Distal cystic duct perforation with small leakage
18	N	Not performed	N	S/p liver transplant
19	Y	Y	Y	Peritonitis and pelvic abscess due to bile leak

## Discussion

Using the standard dosage of Eovist with MRI is efficacious for the detection of bile duct leak after hepatopancreaticobiliary surgery, with a reported sensitivity of 90% and specificity of 74% [10]. Our study provides the first reported data on the use of dEovist for the detection of bile leaks, with a sensitivity of 100% and specificity of 93%. These results provide a working proof of concept and add to the body of research supporting dEovist. Double-dose Eovist has been proven beneficial in other settings as well, including cardiac magnetic resonance for patients with chronic myocardial infarction. Double-dose gadolinium-based contrast agents have been shown to produce improved image quality of scar tissue. When compared with lower doses, the use of double-dose contrast led to higher signal-to-noise and contrast-to-noise ratios, indicating superior image quality [8].

Furthermore, double-dose gadolinium-based contrast-enhanced MRI has proven useful in the delineation of gross tumor volumes of metastatic brain tumors. When compared with single-dose contrast enhancement, double-dose contrast enhancement produced clearer visualization of tumor margins in addition to better enhancement. Doubling the dose of contrast shows a direct correlation with gross tumor volume; when combined with the superior visualization and enhancement of tumor margins seen in these images, double-dose gadolinium-based contrast-enhanced MRI allows for greater precision in stereotactic radiotherapy of metastatic brain tumors [11]. Our findings of high sensitivity and specificity for the detection of bile leaks with dEovist further add to the body of evidence supporting improved image quality with the administration of double doses of Eovist, while also indicating that the modality is highly sensitive and specific for the detection of bile leaks.

Another potential use for dEovist is in patients with hyperbilirubinemia. As previously discussed, HIDA scans are commonly used for the detection of bile leaks. However, HIDA scans are only reliable when the total bilirubin is less than 5 mg/dl. A retrospective study evaluating the reliability of HIDA scans in the detection of biliary complications after liver transplant, including bile leak, obstruction, or stricture, found that HIDA scans performed when the total bilirubin was less than 5 mg/dl had a 100% sensitivity and specificity, but when the total bilirubin was greater than 5 mg/dl, the number of nondiagnostic, inconclusive, and false-negative exams increased [12].

The lower reliability of HIDA scans in patients with hyperbilirubinemia necessitates further research into the use of dEovist for the detection of bile leaks in this population. Following the administration of Eovist, uptake into hepatocytes occurs through organic anion transporting polypeptide 1B3, and excretion occurs through multidrug resistance-associated protein 2 (MRP2) [6]. These are the same transporters used for bilirubin, and it has been shown that hyperbilirubinemia reduces the absorption and excretion of Eovist, leading to suboptimal image quality of the hepatobiliary phase [13]. Given that our present results demonstrate high sensitivity and specificity with a double dosage for the evaluation of bile leaks, further investigation into the potential benefit of doubling the dosage of Eovist to improve image quality in patients with hyperbilirubinemia should be pursued. 

In the evaluation of bile leaks, ERCP is the gold standard. However, as an invasive procedure, there are numerous complications that can occur with ERCP. Systematic reviews have shown that ERCP-attributable complications are relatively common, occurring in 6.85% of cases. These include pancreatitis, bleeding, perforation, and infections [14]. Our results show that dEovist is a promising agent with sensitivity and specificity comparable to ERCP, while carrying a much smaller risk profile. Double-dose Eovist can potentially be an effective diagnostic tool in clinical scenarios where performing ERCP may be technically difficult, dangerous, or impossible. Aside from ERCP, among the current imaging modalities widely utilized to detect bile leaks, MRCP has been shown to have the highest sensitivity and specificity, with sensitivity ranging from 53 to 63% and specificity ranging from 51 to 66%, although it is important to note that these studies have only been conducted post-cholecystectomy [15]. Our study has demonstrated that MRI with double-dose Eovist is not only useful in a wide range of patient presentations, but also demonstrates higher sensitivity and specificity than those reported in MRCP studies.

One group of contrast agents, similar to hepatobiliary-specific agents such as Eovist, that are used for hepatobiliary imaging, includes extracellular fluid agents. Similar to hepatobiliary-specific agents, extracellular fluid agents are gadolinium-based and chelated to an organic compound. Both of these agents circulate freely in the blood and then redistribute to the extracellular fluid space. However, one negative aspect of extracellular agents is their predominantly renal excretion, with approximately 95% renal excretion. There is also the potential for nephrogenic systemic fibrosis, a serious complication of gadolinium contrast agents, especially in patients with preexisting renal conditions. Eovist has a much lower renal excretion percentage, approximately 50% renal excretion, and is associated with the fewest reported cases of nephrogenic systemic fibrosis [16]. Current guidelines from the Radiological Society of North America state that the risk of nephrogenic systemic fibrosis from Eovist is extremely low, as nephrotoxicity is described more commonly with CT iodinated contrast rather than gadolinium MRI contrast [17]. Risks and benefits should be considered for every patient with renal impairment when contrast administration is contemplated. 

There are several limitations to this study, primarily the lack of a control group using the standard single dose of Eovist contrast. Identifying and analyzing a group of patients with a potential bile leak who underwent MRI with a single dose of Eovist would have enabled a direct comparison of the modalities and provided a stronger indication for using dEovist rather than standard-dose Eovist. Secondly, our sample size was relatively small. The retrospective nature of the study resulted in selecting patients with a high probability of bile leaks and does not provide insight into the rate of incidental findings of bile leaks in patients receiving Eovist for other indications. Our study shows promising results, and further research could be conducted in the future with a larger patient population to further analyze the efficacy of dEovist in detecting bile leaks. 

## Conclusions

This study demonstrates that MRI with dEovist offers high sensitivity and specificity for detecting bile leaks, representing a promising noninvasive alternative to current diagnostic approaches. While early identification and treatment of bile leaks are crucial for preventing serious complications, current noninvasive imaging modalities remain limited. ERCP, the current gold standard, is effective but invasive and is associated with significant risks, including bleeding, perforation, infection, and pancreatitis. Our findings highlight the potential of MRI with a double dose of Eovist as a novel noninvasive diagnostic tool for detecting bile leaks following liver transplantation, hepatobiliary surgery, abdominal trauma, and other causes. In a population of patients evaluated for suspected biliary leakage, MRI with a double dose of Eovist consistently identified true bile leaks, with findings confirmed by ERCP or clinical follow-up. The role of MRI with dEovist warrants further investigation on a larger scale to support broader integration of this technique into the diagnosis and management of bile leaks.
